# Koch's Cure

**Published:** 1890-11-22

**Authors:** 


### The Hospital

A Weekly Journal of

Science, Medicine, Nursing, and Philanthropy.

*For Hospitals, Asylums, Infirmaries, Dispensaries, Medical Practitioners, Students, Nurses, and the Charitable Public.*

Vol. IX.—No. 217.] [November 22, 1890.

### Koch's Cure: its Nature and its Limits.

On Friday, November 14th, Dr. Robert Koch published, in the *Deutsche Medizinische Wochenschrift*, an article entitled "A Further Communication on a Remedy for Tuberculosis." The lay press has commented on this article in language of indiscriminate eulogy, and against that we have nothing to say. But at the present moment the medical press has a different task to perform. It does not belong to the crowd who throw their hats into the air and shout "hurrah"; its functions are critical and judicial. Like the judge on the bench and the jury in the box, it must now consider the evidence which is put before it, and must deliver a verdict in strict accordance with the facts. That verdict may be one of approval or of disapproval, or, more probably at the present juncture, of hopeful expectancy.

"As regards the origin and the preparation of the remedy I am unable," says Dr. Koch, "to make any statement, as my research is not yet concluded; I reserve this for a future communication." This is both satisfactory and unsatisfactory—satisfactory in that Dr. Koch intends to make known the nature of his remedy at some future time; unsatisfactory in that having reached a stage at which he considers he has obtained a definite cure for phthisis, he does not make known its nature and the method of its preparation at once. Men accustomed only to the prevalent customs of ordinary business see nothing but merit in a secresy and a monopoly which enable a discoverer to gain wealth and fame at a stroke. But medicine has always laid claim to an entirely different set of motives. It works for mankind first, and for wealth and fame subordinately. If Dr. Koch has produced a positive cure for consumption, the worthiest traditions of medicine demand that he shall not delay by a single hour the giving of every possible facility for the application of such a priceless remedy in every country and in every afflicted home. Moreover, by the immediate communication of his secret, thousands of other skilled brains and fingers would be set to work, not only to use the remedy in practice, but to improve upon it if possible. Just as a noble religion longs most of all for the immediate conversion of every human being to its purity, its faith, and its hope, so does the noblest instinct of medicine demand for its highest satisfaction the speediest possible restoration of every afflicted human being to health and happiness.

Turning now to the scientific rather than the philanthropic aspect of the new discovery, we wish to call attention to certain facts which demand intelligent consideration at the hands of medical critics. Dr. Koch makes a strong point of the fact that the new remedy does not kill the tubercle bacilli but the tuberculous tissue. "It can only," he says, "influence living tuberculous tissue." Before dealing further with this point, we may be permitted to express our regret that the remedy does not kill the tubercle bacilli. In ordinary warfare a besieging army which captures a town and leaves the garrison inside, full-armed, full-fed, and at liberty, has little to boast of. If the bacilli, which are the cause of tubercle, are left in undiminished vigour and activity after the use of the remedy for an indefinite period, the cure will be, to say the least, not so complete as could be desired.

But Dr. Koch claims for his remedy what he calls a "specific anti-tuberculous action"; and he grounds his conviction of this specific action on the appearances presented by lupus spots in patients who have been made the subjects of his treatment. Now this is the crux of the whole subject; and it is to this question of the antituberculous action of the new remedy that medical critics must devote the best powers of their minds. One general fact in the history of universal medicine is here very pertinent. It is this: That true and proper "specifics" for definite forms of disease are almost unknown. We are not forgetting the action of iodide of potassium and other well-known remedies which might be named; but we repeat that true and proper specifics are almost as rare in medicine as snow at the equator, or palm trees at the North Pole. Is it not obvious, therefore, that when any man brings forward a remedy for any disease, but especially for such a disease as consumption, and claims that that remedy is a specific, he makes a large demand upon the faith and practice of intelligent medical men? We do not say that such a demand is always to be opposed; but we do say that those very few drugs which lay claim to specific action have not been established in their position by the researches and authority of a single man. Loyalty to medicine, and to that sensitive conscience which is the true antiseptic and preserver of medicine, demands of all men at this moment the remembrance of past facts in the history of specifics, and the exercise of the clearest judgment on the facts now brought forward for their consideration.

Dr. Koch bases his belief in the "specific anti-tuberculous action" of his new remedy on the appearances seen in lupus spots some time after the remedy has been administered. Now such a fact as this, taken in conjunction with a number of other facts of a convincing nature, would be of much weight. But Dr. Koch practically rests his whole case upon it. Is this fact a foundation of sufficient breadth and solidity to bear the gigantic superstructure which is attempted to be reared thereupon? Dr.

Koch sees certain appearances and interprets them as demonstrating the specific action of the remedy. But is that interpretation the only possible one? There is another, which seems to us, even arguing solely from Dr. Koch's own paper, to be quite as plausible, or perhaps still more plausible. This is the case: The remedy is administered to a patient who has lupus spots upon him. A few hours afterwards the lupus spots begin to visibly swell and redden, and in course of time they pass through certain other stages. It is hence inferred that the drug has a specific action on lupus, and, therefore, on other forms of tubercle. But is it not as probable that the action is not on the lupus spots alone, but on the deeper layers of the skin and subcutaneous tissue of the whole body? It seems to us that it is, and that one fact mentioned by Dr. Koch himself lends great weight to the probability. Among the symptoms which constitute what is called the "general reaction" Dr. Koch mentions that "occasionally an eruption like measles" has appeared on the chest and neck. This is a fact which goes to show, not only that the drug affects the subcutaneous tissue and the deeper layers of the skin, but also in some cases the whole of the skin, and that in a marked degree. If it be asked why we can see the action at the lupus spots and not in other parts of the skin, and why the lupoid tissue is destroyed and shed, whilst the healthy portions of the skin are not manifestly injured, the replies are obvious. At the lupus spots the outer layers of the skin, and sometimes all the layers, have been removed by diseased action, and therefore we can actually see what is going on in the deeper parts; whereas in the healthy portions of the skin the outer layers are all present, and act like a thick stage curtain concealing what is going on behind. With regard to the death and shedding of the lupoid tissue the explanation may be that its death and shedding are due to its previous disease and debility, and its consequent want of power to resist the increased physiological action set up by the drug in the deeper layers of the skin and subcutaneous tissue. That the healthy skin itself does not die is due to the fact that it is healthy and is able to resist the physiological deluge which the energy of the drug has set up. Does a mortified foot ever separate from the body; and does it do so as the result of increased physiological action at the junction of the living and the dead parts? Are the living parts injured by this increased physiological action? And is the whole proceeding due to any specific drug? If it is, what is the name of the drug?

One more point only can we call attention to at the present time. Koch says that, under the treatment, "Patients with cavities not yet too highly developed improved considerably, and were almost cured; only in those whose lungs contained many large cavities could no improvement be proved objectively, though even in these cases the expectoration decreased, and the subjective condition improved. These experiences lead me to suppose that phthisis in the beginning can be cured with certainty by this remedy." The cautiousness of this sentence is worthy of a political lawyer. Three classes of patients who have been treated are mentioned: firstly, those who were "almost cured"; secondly, those in whom "no improvement could be proved objectively"; and thirdly, those who "in the beginning can be cured." We, along with millions of our fellow-creatures, wait for the addition to this list of a fourth class, viz., those who have been cured, definitely and permanently. Until this fourth class is added, and added not on the authority of Dr. Koch alone, or of that of some of his German confrères, but on the proved and repeated experience of competent physicians, in England, France, America, and other civilized countries—until this is done we shall feel compelled to hold ourselves "anxious to believe but hard to be convinced."

